# Hydrometeorological drivers of mosquito (Diptera: Culicidae) abundance in an urbanized region of central Oklahoma

**DOI:** 10.1093/jme/tjag102

**Published:** 2026-07-10

**Authors:** Eric R Bump, Michael C Wimberly

**Affiliations:** Department of Geography and Environmental Sustainability, Sarkeys Energy Center, University of Oklahoma, Norman, OK, USA

**Keywords:** public health entomology, vector ecology, distributed lag models, soil moisture, drought index, solar radiation

## Abstract

Urban mosquito abundance reflects not only temperature and rainfall but also other aspects of the local environment. We quantified species-specific responses to hydrometeorology in Norman, Oklahoma, using two seasons (2023–2024) of weekly data collected with CO_2_-baited BG-Sentinel traps at four sites. Daily meteorological observations were obtained from the Oklahoma Mesonet. The most abundant species were *Aedes albopictus* (Skuse), *Aedes trivittatus* (Coquillett), and the *Culex pipiens* Linnaeus/*Culex quinquefasciatus* Say complex. We modeled environmental relationships using negative binomial generalized additive models that incorporated linear distributed lags (0–56 days) and selected parsimonious combinations of predictors based on AICc. *Ae. albopictus* abundance was associated with shallow soil moisture (5 cm fractional water index) and drought (Standardized Precipitation–Evapotranspiration Index); abundance increased with drier conditions at short lags and with wetter conditions at longer lags. *Ae. trivittatus* was associated with mean temperature, shallow soil moisture, and solar radiation. Abundance increased with drier soils at short lags and with warm, sunny conditions and higher soil moisture at longer lags. *Cx. pipiens/quinquefasciatus* abundance increased with maximum temperature and exhibited a similar relationship with vapor pressure deficit. These results demonstrate that soil moisture, drought indices, and solar radiation capture hydrologic and atmospheric constraints that are often overlooked. Incorporating hydrometeorological variables can improve ecological interpretation and provide actionable indicators for timing urban mosquito control in the southern Great Plains.

## Introduction

Mosquitoes are important vectors of viral, parasitic, and nematode diseases and pose a formidable threat to public health (Vega and Okech 2019, Nebbak et al. 2022). Collectively, vector-borne diseases account for 17% of all infectious diseases and cause around 700,000 human deaths annually (World Health Organization [WHO] 2024). Effective control of disease-transmitting mosquitoes depends not only on pathogen ecology but also on understanding the environmental factors that influence mosquito abundance (LaDeau et al. 2015, Brugueras et al. 2020). Abundance data enable health agencies to anticipate when and where adult populations of key vectors will peak, allowing them to optimize interventions such as larval source control or adulticiding (Yang et al. 2009, Schwab et al. 2018, Garcia-Luna et al. 2019, Ventura et al. 2024). Forecasting mosquito abundance hinges on identifying the environmental drivers that shape vector dynamics (Myer et al. 2017, Kache et al. 2020, O’Dell et al. 2025). Most climate-mosquito models emphasize temperature and rainfall, with fewer incorporating humidity, yet mosquitoes experience a broader suite of interacting environmental conditions that modulate their population dynamics (Yamana and Eltahir 2013, Brugueras et al. 2020, Cazelles et al. 2023). There is growing recognition that focusing solely on temperature and precipitation oversimplifies the complex ecological niche occupied by mosquitoes (Roiz et al. 2014, Smith et al. 2020, Ma et al. 2023, Bump et al. 2025).

Mosquitoes are archetypal ectotherms, and their life cycles are highly sensitive to abiotic conditions (Kumar et al. 2024). Temperature governs rates of development, survival, and fecundity; rainfall supplies water for larval (immature) developmental habitats; atmospheric humidity modulates desiccation stress; and wind influences dispersal and feeding (Shaman et al. 2002, Eisen et al. 2014, Schmidt et al. 2018). Temperature is a dominant driver of ectotherm physiology (Rozen-Rechels et al. 2019). Increasing temperatures accelerate enzymatic reactions up to an optimum, beyond which protein denaturation and desiccation stress cause rapid declines in performance (Schoolfield et al. 1981, Shapiro et al. 2017, Kontopoulos et al. 2018, Schmidt et al. 2018). Numerous studies document nonlinear thermal performance curves for mosquito development, survival, and biting rates, leading to unimodal relationships between temperature and vectorial capacity (Mordecai et al. 2013, 2019, Rezende and Bozinovic 2019, Shocket et al. 2020). Rainfall both creates and flushes larval habitats, so moderate precipitation can enhance mosquito populations, while heavy downpours can wash larvae from small containers (Stewart Ibarra et al. 2013, Caldwell et al. 2021). Temperature and precipitation, therefore, underpin most mechanistic models of mosquito-borne disease (Parham and Michael 2010, Rozen-Rechels et al. 2019).

Humidity is often overlooked despite its biological importance, and even when considered, it is frequently treated independently of temperature, thereby ignoring its synergistic effects (Brown et al. 2023). Relative humidity modulates evaporative water loss, egg desiccation, and adult longevity; empirical studies report that higher humidity improves survival, egg production, and activity of mosquitoes (up to ∼90 % RH), whereas drier conditions suppress these traits but can increase biting rates (Alto and Juliano 2001, Hagan et al. 2018, Holmes et al. 2022, Brown et al. 2023). Theoretical work proposes that temperature and humidity interact to shape thermal performance, yet this interaction is rarely incorporated into transmission models (Yamana and Eltahir 2013). Moreover, in mosquitoes, thermo-hydroregulation requires that heat and water balance must be maintained simultaneously; body temperature and hydration jointly shape survival and behavior, and tradeoffs may arise when humid microhabitats favor water balance but diverge from thermal optima (Kessler and Guerin 2008, Rozen-Rechels et al. 2019, Reinhold et al. 2021).

Other environmental factors are also emerging as important determinants of mosquito-borne disease transmission. Hydrometeorological extremes, including droughts and floods, were linked to dengue risk in Brazil, with extremely wet conditions increasing risk within 1 month and droughts increasing risk after 4 months (Lowe et al. 2021). These effects likely reflect changes in developmental sites and human water-storage behaviors during water shortages, echoing observations from Brisbane, where drought-induced rainwater harvesting created new habitats for container mosquitoes (Trewin et al. 2013). Wind has been neglected in most models, but it can suppress mosquito flight and virus transmission. In West Nile virus models for Louisiana and South Dakota, including wind speed improved model fit, and higher wind speeds were associated with fewer cases (Bump et al. 2025). Soil moisture is crucial for the availability and persistence of larval habitats, particularly in temporary pools and floodwater sites. For *Aedes* species that lay desiccation-resistant eggs on moist substrates, soil moisture can determine hatching success and population dynamics, while drier soils may limit emergence (Armbruster 2016, Ukawuba and Shaman 2018, Holmes and Benoit 2019). Solar radiation influences both aquatic and terrestrial stages, driving evaporation and surface warming of larval habitats, which affect development rates and survival, while also causing desiccation stress in adults active in exposed environments (Villena et al. 2018, Berry et al. 2020). A substantial knowledge gap exists regarding how underexplored environmental variables beyond temperature and rainfall influence mosquito ecology and vector abundance, emphasizing the need to examine their roles in a species- and region-specific context. Mosquito population dynamics are also shaped by the temporal sequence in which conditions unfold, not just the identity of the drivers themselves. A period of soil saturation followed by a brief dry-down, for instance, may trigger emergence pulses that neither variable alone would predict (Shaman et al. 2002, Lowe et al. 2021). Characterizing these sequences and the time lags over which they operate can improve ecological interpretation and inform the timing of vector control interventions.

Oklahoma, located in the southern Great Plains, spans a climatic gradient from humid subtropical conditions in the east to semi-arid conditions in the west, providing an ideal setting to investigate these questions (Oklahoma Climatological Survey 2025). More than 60 mosquito species occur seasonally in Oklahoma, and urban surveillance has identified *Ae. albopictus* and the *Cx. pipiens* complex among the most abundant taxa in city settings (Bradt et al. 2019, Crans 2025). The geographic range of *Ae. albopictus*, a species native to Asia, expanded rapidly following its introduction into the United States, increasing the potential for Aedes-borne arboviral risk; climate change is also expected to redistribute transmission risk for Aedes-borne viruses (Ryan et al. 2019, Hoekman et al. 2022). In Oklahoma, the primary *Culex* vectors associated with major West Nile virus outbreaks have differed from year to year, underscoring that vector–pathogen dynamics can shift over time (Bradt et al. 2019, Hoekman et al. 2022, McMahon et al. 2022). However, ecological studies that explore species-specific responses to a broad range of local climatic and hydrological variables remain limited. Norman sits near the center of Oklahoma’s east–west climatic gradient in the humid subtropical zone, where warm summers, variable precipitation, and periodic drought produce pronounced hydrometeorological variability within and across seasons. Combined with the high-quality daily environmental data available from the Oklahoma Mesonet, these conditions make Norman a productive site for examining species-specific responses to a broad suite of environmental drivers.

The objectives of this study were to: (1) quantify how daily mosquito abundance across multiple species responds to a broad suite of meteorological and hydrometeorological variables at short to medium time lags, including the temporal sequences of conditions that precede abundance increases; (2) assess whether variables beyond temperature, precipitation, and relative humidity improve model performance; and (3) identify species-specific environmental sensitivities to inform targeted surveillance and control strategies in the southern Great Plains. To achieve these aims, we developed statistical time series models of mosquito abundance using meteorological data. By addressing these questions, our study advances efforts to integrate thermal, hydrological, and atmospheric processes into mosquito ecology, thereby enhancing the modeling of mosquito abundance dynamics.

## Methods

### Study Area

**Fig. 1.**
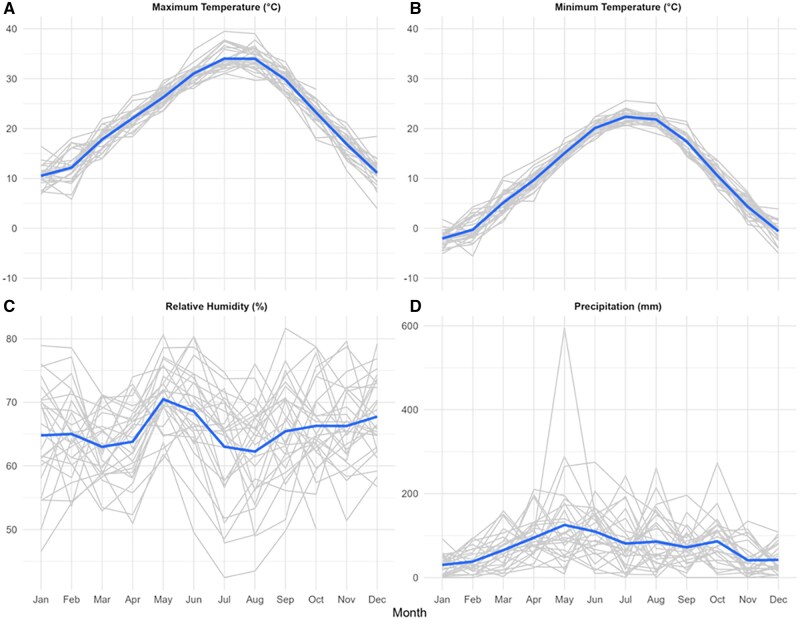
Seasonal climate by month for Norman, OK (1997–2024): (A) maximum temperature (°C), (B) minimum temperature (°C), (C) relative humidity (%), and (D) precipitation (mm). Thin grey lines show each individual year; the bold blue line is the multi-year monthly mean (climatology). All weather variables are computed from daily values.

Norman is a mid-sized city located in Cleveland County, Oklahoma (approximately 35.2° N, 97.4° W), with a population of 128,026 and an area of about 463 km^2^ (U.S. Census Bureau 2024). The city lies on the southern edge of the Oklahoma City metropolitan area in the southern Great Plains. The climate is characterized as humid subtropical; summers are hot and humid, while winters are generally cool, with occasional brief cold snaps (Oklahoma Climatological Survey 2025). Monthly mean daily maximum temperatures range from approximately 10.5 °C in January to 34.0 °C in July, while monthly mean daily minimum temperatures range from −2.1 °C to 22.4 °C, respectively. Mean annual precipitation is 874 mm, with the wettest month typically being May (∼125 mm) and the driest January (∼31 mm). The annual mean relative humidity is ∼65.6%, with values peaking in spring (e.g., ∼70.5% in May) and dropping off during the hottest months, reaching their lowest monthly averages in July and August (∼62–63%) (Fig. 1) (Brock et al. 1995, McPherson et al. 2007).

### Field Sampling and Mosquito Identification

**Fig. 2.**
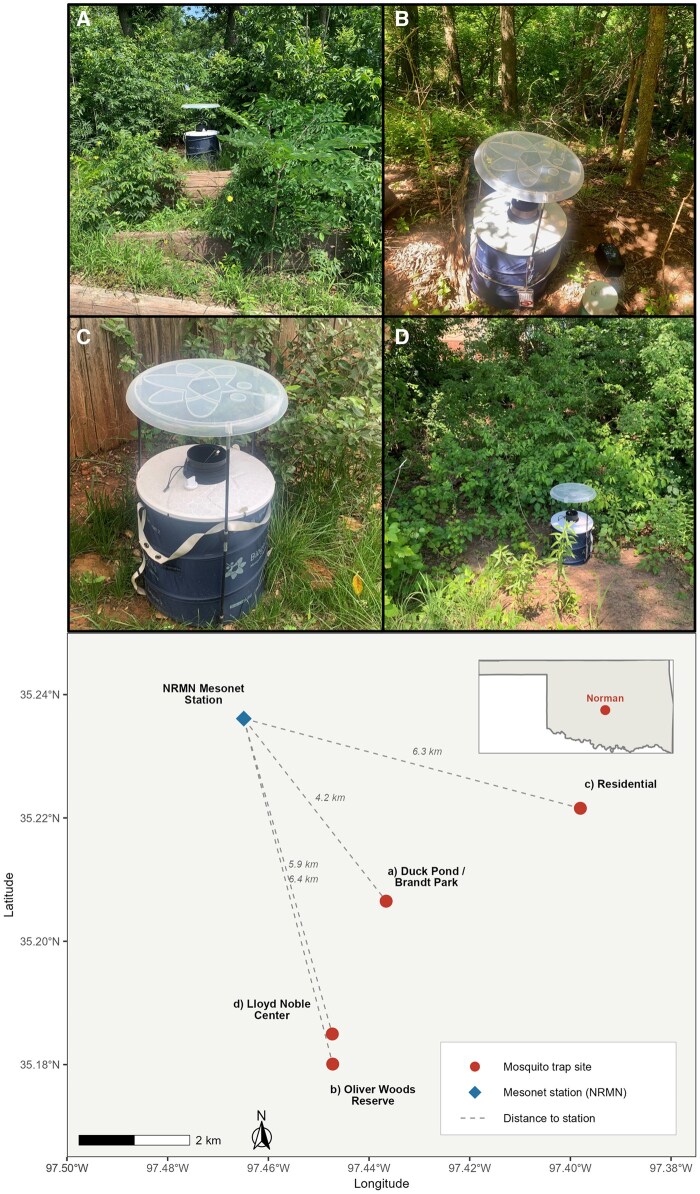
Study area in Norman, Oklahoma. Map (bottom) showing the four mosquito trap sites (red circles) relative to the Oklahoma Mesonet station at Max Westheimer Airport (blue diamond), the source of the meteorological data used in this study; dashed lines connect each trap site to the station and are annotated with the approximate distance between them. Trap site photographs (top): (A) Duck Pond/Brandt Park, an urban park on the OU campus with a permanent water body and high pedestrian activity; (B) Oliver Woods Reserve, a mixed woodland preserve; (C) a residential backyard in East Norman; and (D) a riparian site near a stormwater drain and creek adjacent to the Lloyd Noble Center, a large athletic complex with surrounding parking lots.

Land cover across the study area transitions from urban and suburban development surrounding the University of Oklahoma (OU) campus to floodplain woodlands and agricultural lands near the Canadian River (Penfound 1948, Oklahoma Biological Survey 2025). To capture this environmental heterogeneity, we selected four distinct trap sites (Fig. 2): (1) Oliver Woods Reserve, a mixed woodland preserve; (2) Duck Pond/Brandt Park, a landscaped urban park featuring a permanent water body and high pedestrian activity on the OU campus; (3) near a creek adjacent to a stormwater drain near the Lloyd Noble Center, a large athletic complex bordered by expansive parking lots; and (4) a residential backyard in East Norman.

Mosquitoes were collected twice weekly at each trap site during two consecutive field seasons. In Year 1 (May 30–October 28, 2023) and Year 2 (April 9–October 31, 2024), traps were deployed, with each deployment lasting 48 h across two consecutive days. This schedule yielded 52 trapping weeks and 104 trap collections per site. Mosquitoes were captured using BG-Sentinel™ traps (Biogents AG, Regensburg, Germany) baited with a commercial BG-Lure and approximately 1 kg of dry ice; the CO_2_ source was replenished after 24 h. The BG-Sentinel is a lightweight, collapsible monitoring trap that requires only a 12 V DC power source (Biogents AG 2025). Its bidirectional convection currents mimic the heat exchange and convection of a human body, making it particularly effective against *Aedes aegypti* and *Aedes albopictus*, while also attracting *Culex* species (Bidlingmayer et al. 1995, Farajollahi et al. 2009, Pezzin et al. 2016, Biogents AG 2025). Traps were positioned approximately 1 m above the ground in shaded locations and protected from rainfall using weather shields.

Each trap was operated in a single 48-h session per week and serviced at ∼24 and ∼48 h after deployment, with the CO2 source replenished at the 24-h servicing, producing two consecutive ∼24-h collections per session. Upon retrieval, the collected specimens were transported to a laboratory on the OU campus in insulated containers and stored at −80 °C until processing (Centers for Disease Control and Prevention 2024). Adult mosquitoes were sorted, sexed, and identified to species using morphological keys under a stereomicroscope. Morphological identification remains the reference method for mosquitoes because it requires little equipment, is inexpensive, and is broadly applicable to operational surveillance (Jourdain et al. 2018). Identification was based on dichotomous keys, with ambiguous samples resolved to species complexes (e.g., *Culex pipiens*/*quinquefasciatus*) (Bradt et al. 2017). For each site, species, and collection day, we recorded the total number of adult females. Each trapping session spanned two consecutive days, and the two ∼24-h collections were retained as separate observations , preserving daily resolution for the distributed lag analysis. Each daily count therefore constituted one observation in the subsequent statistical models, yielding two observations per site per trapping week and 416 site-date observations per species across the four sites and both seasons (4 sites × 104 collection days).

### Species Focus

**Fig. 3.**
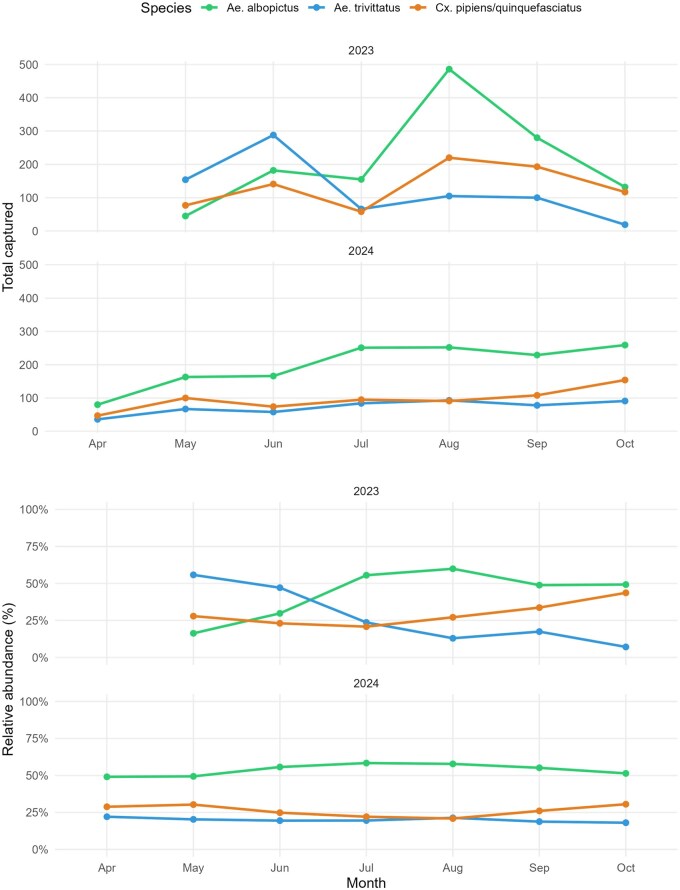
Monthly mosquito totals and relative abundance (Apr–Oct). The top two panels show the total captures per month, aggregated across traps, for 2023 (top) and 2024 (bottom). The bottom two panels show the relative abundance (%) of the same three taxa for 2023 and 2024, respectively. Lines correspond to the different species, *Ae. albopictus*, *Cx. pipiens*/*quinquefasciatus*, and *Ae. trivittatus* as indicated in the legend.

Across the 2023–2024 campaigns, we collected mosquitoes representing at least 15 species. Captures were highly uneven, with three taxa comprising the majority of specimens (Table 1). The most frequently collected taxa were *Aedes albopictus*, the *Culex pipiens/quinquefasciatus* complex, and *Aedes trivittatus*; *Psorophora ferox* was the next most common, and all remaining taxa were comparatively rare (Table 1). This distribution informed our modeling strategy: to maximize statistical power and focus on medically important taxa, we analyzed counts for the three most abundant taxa (Fig. 3).

**Table 1.** Abundances of mosquito species captured in Norman, Oklahoma, during two consecutive field seasons (2023–2024)

Species	2023	%	2024	%	Total	%
***Aedes albopictus***	1,280	28.23	1,400	37.53	2,680	32.43
***Culex pipiens/quinquefasciatus***	806	17.78	669	17.94	1,475	17.85
***Aedes trivittatus***	732	16.14	507	13.59	1,239	14.99
***Psorophora ferox***	452	9.97	230	6.17	682	8.25
***Culex nigripalpus***	199	4.39	126	3.38	325	3.93
***Aedes vexans***	205	4.52	104	2.79	309	3.74
***Culex salinarius***	149	3.29	89	2.39	238	2.88
***Aedes triseriatus***	154	3.40	83	2.23	237	2.87
***Culiseta inornata***	114	2.51	79	2.12	193	2.34
***Culex tarsalis***	122	2.69	68	1.82	190	2.30
***Psorophora columbiae***	80	1.76	68	1.82	148	1.79
***Psorophora cyanescens***	78	1.72	64	1.72	142	1.72
***Anopheles punctipennis***	54	1.19	84	2.25	138	1.67
***Anopheles pseudopunctipennis***	45	0.99	79	2.12	124	1.50
***Anopheles quadrimaculatus***	22	0.49	50	1.34	72	0.87
**Unknown**	42	0.93	30	0.80	72	0.87
**Total**	4,534	100.00	3,730	100.00	8,264	100.00

Counts represent the total number of adults collected in BG-Sentinel traps across all four study sites. Abundance was highly skewed, with *Ae albopictus.*, *Cx. pipiens/quinquefasciatus*, and *Ae. trivittatus* accounting for most captures, while the remaining species were observed at substantially lower frequencies. Relative abundances (%) represent the proportion of total captures attributed to each species within a given year and across both years combined.

### Meteorological and Hydrometeorological Data

Daily meteorological data were extracted from the Oklahoma Mesonet station at Max Westheimer Airport. The Mesonet was commissioned in 1994 and consists of at least one automated station in each of Oklahoma’s 77 counties (Brock et al. 1995, McPherson et al. 2007). Each station employs sensors mounted on a 10 m tower that measure environmental variables every 5 min (Brock et al. 1995, McPherson et al. 2007). The Norman (NRMN) Mesonet station, located at Max Westheimer Airport in northwestern Norman, approximately 4–7 km from the trap sites, provided spatially representative meteorological data for the study area. Daily meteorological data (1997–2024) were obtained from the Oklahoma Mesonet. They included daily maximum air temperature, daily minimum air temperature, daily mean air temperature, mean dew point temperature, mean relative humidity, mean vapor pressure deficit, total precipitation, mean wind speed at 10 m and 2 m, and daily accumulated solar radiation (Brock et al. 1995, McPherson et al. 2007). To evaluate whether the Mesonet station adequately represented conditions at the trap sites, we deployed SensorPush wireless temperature and relative humidity data loggers (SensorPush, Inc., Brooklyn, NY) at each of the four trapping locations during both field seasons (2023–2024). At each site, two loggers were placed: one in a shaded position and one in an exposed location. Loggers recorded temperature (°C) and relative humidity (%) at 1-min intervals throughout the trapping period. For each site and date, the shaded and exposed logger readings were averaged to produce a single daily mean temperature and relative humidity value. These daily values were then aggregated to ISO-week means and compared with the corresponding weekly means from the Mesonet station (Supplementary Fig. S1). Temperature and humidity tracked closely between the two data sources, supporting the use of the single Mesonet station as the primary meteorological data source for the models.

Other weather variables were derived from these primary meteorological observations. Specific humidity was approximated from daily mean temperature and relative humidity using standard psychrometric relations (Parish and Putnam 1977). Although this approach may smooth subdaily variability compared with computing specific humidity from higher-frequency (e.g., 5-min) observations and then averaging, we expect any resulting differences in daily values to be modest relative to the temporal scales considered in our weekly lagged analyses. Seven-day accumulated precipitation was computed using a rolling window with ≤2-day linear gap-filling, and the resulting totals were log-transformed to stabilize variance.

Drought stress was characterized using the Standardized Precipitation–Evapotranspiration Index (SPEI) at a one-month timescale. To compute monthly SPEI values, we aggregated total precipitation and reference evapotranspiration (ETOS) over each calendar month, calculated the water balance (precipitation minus ETOS), and fitted a three-parameter log-logistic distribution to the historical distribution of monthly water balances. The cumulative distribution was then transformed to a standard normal *z*-score (Vicente-Serrano et al. 2010, Beguería et al. 2014, Lee et al. 2024). The resulting monthly SPEI values were assigned to each day within the corresponding month and winsorized to ±3 to reduce the influence of extreme values.

Reference evapotranspiration was calculated using the FAO-56 Penman–Monteith equation, a method widely regarded as the global standard for ET estimation due to its physical basis and consistent performance across diverse climates. We used the short-crop version of ET (ETOS), which assumes a standardized, well-watered, clipped grass canopy approximately 0.12 m tall with fixed aerodynamic and physiological parameters (Food and Agriculture Organization of the United Nations 2025). This version is favored in large-scale drought indices because it minimizes spatial heterogeneity across vegetation types, enabling comparisons across ecosystems (Zotarelli et al. 2024).

We also derived Fractional Water Index (FWI) values to represent soil moisture conditions at depths of 5 cm and 25 cm. These were calculated from soil temperature response sensors TR05 and TR25, which are part of the Oklahoma Mesonet’s soil monitoring system. Following Illston et al. (2008), the FWI is a normalized indicator of plant-available soil moisture derived from heat dissipation sensors (Campbell 229-L). The FWI is computed as:


$$\mathrm{FWI}=\;\frac{\Delta T_{d}-\;\Delta T_{\mathrm{ref}}}{\Delta T_{d}-\;\Delta T_{w}},$$

where $\Delta T_{d}$ = 3.96 °C, $\Delta T_{w}$ = 1.38 °C, and Δ*T*_ref_ is the daily measured temperature difference. The FWI is unitless and ranges from 0.0 (very dry) to 1.0 (field capacity), making it insensitive to local soil texture and ideal for mesoscale analyses (Illston et al. 2008). All variables and their transformations are summarized in Table 2.

**Table 2.** Meteorological and hydrometeorological variables used in the analysis

Variable	ID	Unit	Conceptual family
**Daily maximum air temperature**	TMAX	°C	Temperature
**Daily minimum air temperature**	TMIN	°C	Temperature
**Daily mean air temperature**	TAVG	°C	Temperature
**Daily mean dew‑point temperature**	DAVG	°C	Humidity
**Daily mean relative humidity**	HAVG	%	Humidity
**Daily mean vapor pressure deficit**	VDEF	mbar	Humidity
**Specific humidity**	SH	g kg−¹	Humidity
**Fractional water index at 5 cm soil depth**	FWI05	0–1 (dimensionless)	Soil moisture
**Fractional water index at 25 cm soil depth**	FWI25	0–1	Soil moisture
**Seven‑day accumulated precipitation**	RAIN7_log	log(mm+1)	Rainfall
**Standardized precipitation–evapotranspiration index (1‑month)**	SPEI_w	*z*‑score	Drought
**Mean wind speed at 10 m**	WSPD	m s−¹	Wind
**Mean wind speed at 2 m**	W2S	m s−¹	Wind
**Total solar radiation**	ATOT	MJ m−² day−¹	Solar radiation

Meteorological data were obtained from the Oklahoma Mesonet station at Max Westheimer Airport. Most variables represent daily means, maxima, or minima; others (e.g., RAIN7_log, SPEI_w) are multi-day or monthly indices derived from these daily observations, as described in the Methods. Transformations and derivations are indicated in the table. The “Conceptual family” column indicates the variable groupings used in the multimodel selection procedure, in which at most one variable per family was allowed in each candidate model.

### Research Design and Modeling Rationale

The core analytical goal was to quantify how 48-h mosquito counts (collected at weekly intervals) respond to weather and hydrometeorological variability across short- to medium-term lag windows (0–56 days) (Manica et al. 2020, Lim et al. 2021). Because environmental conditions can delay mosquito development and survival, we used a linear distributed lag model (DLM) framework embedded within a generalized additive modeling (GAM) structure (Gasparrini et al. 2010, Gasparrini 2011, Hastie 2017).

DLMs allow a predictor’s effect to unfold over a specified lag period; when implemented with a cross-basis, they can simultaneously capture the exposure–response and lag–response functions (Gasparrini et al. 2010, Gasparrini 2011). In our implementation, the exposure–response relationship for each environmental variable was assumed to be linear, and the lag dimension was represented by a series of linearized lag terms covering 0–56 days. That is, we included a separate linear term for exposure at each lag day (e.g., temperature 0 days before sampling, 1 day before, 2 days before, and so on up to 56 days). This specification estimates a distinct effect for each lag, assuming that changes in exposure at each lag have a linear effect on mosquito abundance. We did not impose additional smoothing across lags, which allows the timing and shape of delayed effects to be driven directly by the data. Each of the three focal species was modeled separately. Observations consisted of 48-h trap counts at each site on each collection date; all four sites were included in the same model (416 site-date observations per species), with among-site heterogeneity captured by a site-level random intercept. The site-level random intercept accounts for the nonindependence of observations collected repeatedly at the same location by allowing baseline abundance to vary among sites. Temporal structure was addressed through two additional model components: a cyclic smooth of day of year captured seasonal trends in abundance and a distributed lag cross-basis that conditioned each observation on the preceding 56 days of meteorological conditions, thereby absorbing much of the temporal dependence in the data. We did not impose an explicit residual autocorrelation structure [e.g., AR (1)], as the combination of weekly sampling intervals, the seasonal smooth, and the extended lag window substantially reduces residual temporal autocorrelation.

### Single Variable Analyses

For each species, we first fitted univariate models to examine daily trap counts as a function of each of the 14 environmental variables. In each single-variable model, the predictor was entered via the linear cross-basis described above, spanning lags from 0 to 56 days. Practically, this means the model estimated a separate linear effect for exposure at each lag day, conditional on exposures at the other lag days. Although trap counts were aggregated over 48-h deployment periods, the meteorological predictors were entered into the model as daily values, and weather variability within each deployment period was represented at daily resolution through the distributed lag structure.

The outcome was the daily trap count at each site, and overdispersion was addressed by choosing a negative binomial response distribution (Heersink et al. 2016, Manica et al. 2020). Seasonal structure was captured by a cyclic smooth of day of year, and among-site heterogeneity was modeled with site-level random intercepts (Yoo 2014). Exposures were centered (e.g., soil moisture at 0.5 and the drought index at 0) to aid interpretation of relative risks.

After fitting each model, we extracted the estimated relative risk surface (exposure × lag), presented as a contour plot and as slice plots showing lag–response curves at representative quantiles and exposure–response curves at selected lags. Relative risk compares the predicted incidence (in our case, mosquito abundance) under a given exposure–lag combination to that under a reference level; a value of 1 indicates no change, values greater than 1 indicate higher abundance relative to the reference, and values less than 1 indicate lower abundance. This step allowed for a visual assessment of the timing and magnitude of delayed effects for each weather variable. Cumulative and lag-specific RR estimates for the selected models are summarized in Supplementary Table S1.

### Multimodel Inference and Selection Strategy

Mosquitoes typically respond to multiple environmental factors, so we also considered additive multivariable models and interpreted them within an information-theoretic, multimodel inference framework (Burnham and Anderson 2002). To limit collinearity and retain interpretability, variables were grouped into conceptual families (temperature, humidity, wind, soil moisture, rainfall, drought, and solar radiation), and at most one variable per family was allowed in each model. All unique combinations of one to four predictors satisfying this “one per family” rule were evaluated. Each candidate model used the same distributed lag and seasonal structure as the univariate analyses, and model fit was summarized using the corrected Akaike Information Criterion (AICc) (Cavanaugh 1997). AICc adjusts for small sample sizes by penalizing models with more parameters more heavily than the standard AIC; this is particularly important when the number of observations is modest relative to the model's potential complexity.

For each species, we ranked models by AICc and treated models with ΔAICc ≤ 2 as a supported set of competing models. Within this set, we followed recommendations from the multimodel inference literature to be cautious about uninformative parameters (Arnold 2010, Leroux 2019), that is, predictors that appear in more complex models but add little to model fit and have small, highly uncertain effects. When a more complex model was a nested extension of a simpler one and differed only by such uninformative terms, we emphasized the simpler, more parsimonious model in our interpretation; when additional predictors had clear, interpretable effects (as in the *Ae. trivittatus* model), we retained the more complex model even if simpler alternatives had similar AICc.

When several supported models were not simply nested versions of one another, we considered both parsimony and Akaike weights (*W_i_*) when deciding which model to highlight. For each species, we present, in Table 3, the “best” model as the candidate with the lowest AICc and a relatively high *W_i_* that also provided a biologically interpretable combination of predictors, while noting that alternative, simpler models within the ΔAICc < 2 set also retained appreciable support. This two-stage procedure, univariate exploration followed by multivariable selection under a multimodel inference framework, provides a transparent and reproducible way to identify parsimonious combinations of weather drivers without over-interpreting weak or uninformative effects.

**Table 3.** Model selection results for *Aedes albopictus*, *Aedes trivittatus*, and *Culex pipiens/quinquefasciatus* based on distributed-lag models with a 56-day window

Species	Model	AICc	ΔAICc	W_i_
**Aedes albopictus**	**SPEI + FWI05**	1,899.85	0.00	0.33
	SPEI + TMAX	1,901.47	1.62	0.14
	SPEI + FWI25	1,901.48	1.63	0.14
	SPEI + TMIN + VDEF	1,901.60	1.75	0.14
	SPEI + FWI05 + ATOT	1,901.67	1.82	0.13
	SPEI + VDEF	1,901.87	2.00	0.12
**Aedes trivittatus**	**ATOT + TAVG + FWI05**	1,136.35	0.00	0.16
	ATOT + TMIN	1,137.24	0.89	0.10
	ATOT + TMIN + FWI05	1,137.28	0.93	0.10
	ATOT + DAVG	1,137.40	1.06	0.09
	ATOT	1,137.42	1.07	0.09
	ATOT + TMAX	1,137.63	1.28	0.08
	ATOT + TMIN + RAIN7_log	1,137.80	1.46	0.07
	ATOT + HAVG	1,138.10	1.75	0.06
	ATOT + TAVG + RAIN7_log	1,138.13	1.79	0.06
	ATOT + TMAX + WSPD + FWI05	1,138.19	1.85	0.06
	ATOT + TAVG + WSPD + FWI05	1,138.23	1.89	0.06
	ATOT + SH	1,138.35	2.00	0.06
**Culex pipiens/quinq.**	**TMAX**	1,474.82	0.00	0.50
	VDEF	1,475.77	0.95	0.31
	SPEI + TMAX	1,476.76	1.94	0.19

Candidate models include univariate and multivariate combinations of climate variables, constrained to one variable per conceptual family. Models are ranked by small-sample corrected Akaike Information Criterion (AICc) and accompanied by Akaike weights (*W_i_*). For each species, models with ΔAICc ≤ 2 were treated as a supported set of competing models. Within this set, the most parsimonious model with relatively large Akaike weight is highlighted in blue and used as the primary model in the text; other models shown also retain competitive support.

## Results

We evaluated linear distributed lag models with a cyclic seasonal smooth for each of the three mosquito species, comparing all allowable combinations of meteorological and hydrometeorological predictors. For each species, candidate models were ranked by AICc, and models with ΔAICc ≤ 2 were considered a supported set of competing models (Table 3). Within this set, we selected as the “best” model the candidate with the largest Akaike weight (*W_i_*); when two or more models had similar *W_i_*, we favored the more parsimonious and biologically interpretable combination of predictors, while recognizing that alternative models within the ΔAICc ≤ 2 range also retained appreciable support. Cumulative and lag-specific relative risk estimates for each predictor in the selected models are reported in Supplementary Table S1.

### Aedes albopictus

Using Akaike weights, the model pairing shallow soil moisture at 5 cm (FWI05) with drought (SPEI) had the greatest support (*W_i_* = 0.33; AICc = 1,899.85). Several alternatives were within ΔAICc ≈ 1.6–2.0 but had smaller weights (e.g., SPEI + TMAX *W_i_* = 0.14, ΔAICc = 1.62; FWI25 + SPEI, *W_i_* = 0.14, ΔAICc = 1.63; SPEI + TMIN + VDEF, *W_i_* = 0.14, ΔAICc = 1.75). Notably, all competing models with ΔAICc < 2 included SPEI, suggesting a recurring contribution of drought conditions to model fit. We therefore selected SPEI + FWI05 as the final model for interpretation.

**Fig. 4.**
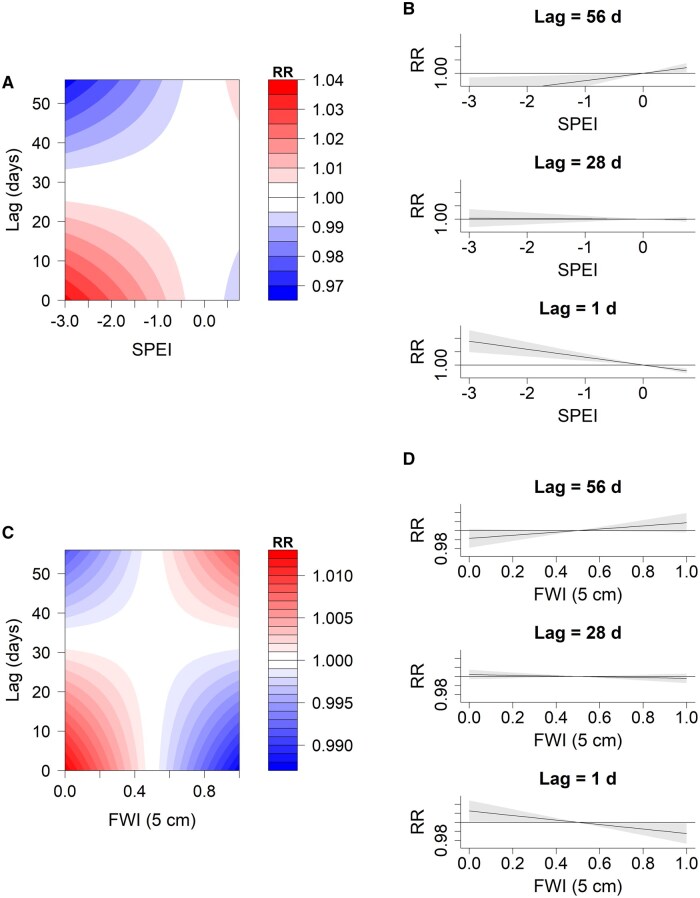
Exposure-lag associations for Aedes albopictus (Diptera: Culicidae) from the selected distributed-lag model, including drought stress (SPEI) and shallow soil moisture (FWI05). SPEI = Standardized Precipitation–Evapotranspiration Index (1-month standardized z-score; negative values indicate drier-than-normal conditions and positive values wetter-than-normal). FWI05 = Fractional Water Index at 5-cm soil depth (unitless; 0 = very dry, 1 ≈ field capacity). RR = relative risk (RR = 1 indicates baseline; RR > 1 higher abundance; RR < 1 lower abundance). A, C) Contour plots of RR surfaces across exposure values and lags (0–56 d) for SPEI and FWI05, respectively. B, D) Fixed-lag slice plots (SPEI and FWI05, respectively) showing RR versus exposure at lags of 56 d (top row), 28 d (middle row), and 1 d (bottom row).

To interpret the results of the selected model for *Ae. albopictus* (SPEI + FWI05), we examined the corresponding exposure–lag surfaces (Fig.  4A–D). The FWI05 surface showed a cross-over pattern: at short lags (≈0–10 days), drier soils (low FWI05) were associated with elevated relative risk (RR > 1), whereas wetter soils were associated with reduced risk. At longer lags (≈approximately 40–56 days), the relationship reversed, with wetter soils associated with a higher risk and drier soils with a lower risk. Effects around intermediate lags (≈20–30 days) were near null. The slice plots highlighted this transition. RR declined with increasing FWI05 at a 1-day lag, was minimal at 28 days, and increased with FWI05 at 56 days. Strong drought (SPEI ≈ –2.0) was linked to elevated relative risk at short lags (< 10 days), declining to baseline by 35–56 days; milder drought (≈ –1.25 to –0.25) showed smaller, similarly declining effects. In contrast, high SPEI lowered the relative risk to below 1.0 at short lags but raised it above 1.0 at longer lags. Slice plots illustrated this timing: the SPEI–risk association was strongest at 1 day, flattened by 28 days, and remained weakly positive by 56 days.

### Aedes trivittatus

The model that included ATOT, TAVG, and FWI05 (*W*_i_ = 0.16; AICc = 1,136.35) had the lowest AICc and the largest Akaike weight. Several simpler models were also well supported (ΔAICc ≤ 2) but carried lower weights, including ATOT + TMIN (*W_i_* = 0.10, ΔAICc = 0.89), ATOT + DAVG (*W_i_* = 0.09, ΔAICc = 1.06), ATOT alone (*W_i_* = 0.09, ΔAICc = 1.07), ATOT + TMAX (*W_i_* = 0.08, ΔAICc = 1.28), and ATOT + HAVG (*W_i_* = 0.06, ΔAICc = 1.75; Table 3). The presence of ATOT in all competitive models suggests that solar radiation is a robust predictor of *Ae. trivittatus* abundance, whereas the choice of accompanying temperature and moisture terms is less certain. Consistent with our model-selection strategy, we highlight ATOT + TAVG + FWI05 as the primary model because it combines relatively high support with a biologically interpretable set of predictors; the additional TAVG and FWI05 terms showed consistent, interpretable associations with abundance, so we treated this as the most informative model within the supported set.

**Fig. 5.**
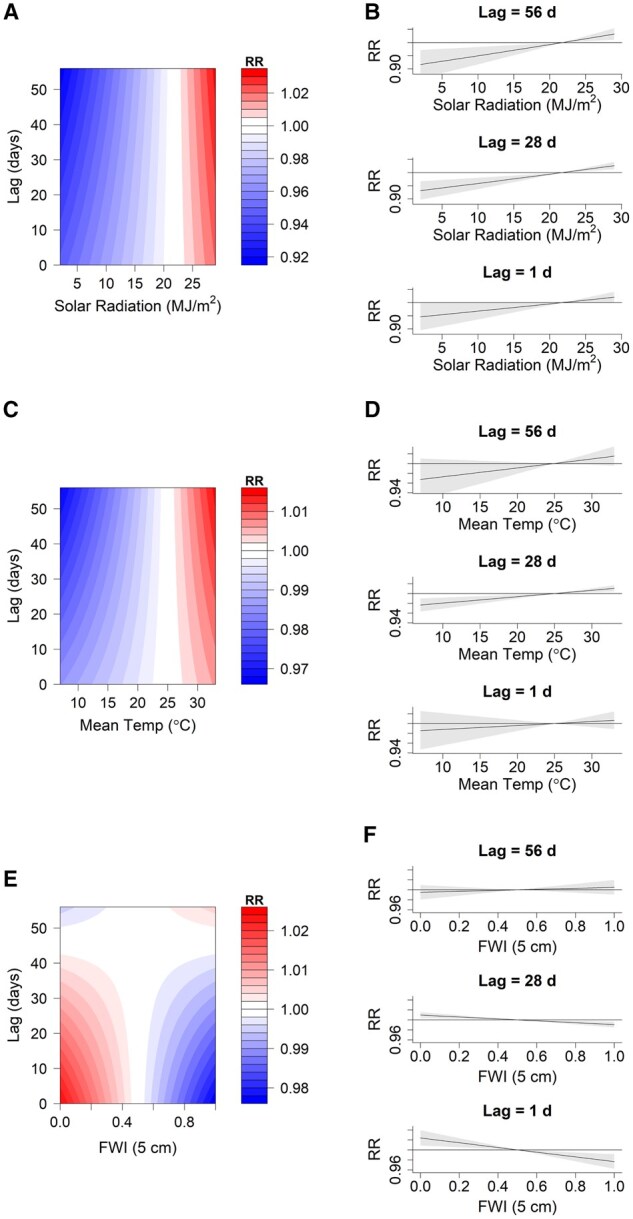
Exposure–lag associations for Aedes trivittatus (Diptera: Culicidae) from the selected distributed-lag model including total solar radiation (ATOT), mean air temperature (TAVG), and shallow soil moisture (FWI05). ATOT = total solar radiation (MJ m−² day−¹); TAVG = daily mean air temperature (°C); FWI05 = fractional water index at 5-cm soil depth (unitless; 0 = very dry, 1 ≈ field capacity); RR = relative risk (RR = 1 indicates the reference level; RR > 1 higher abundance; RR < 1 lower abundance). Panels (A), (C), and (E) show contour plots of RR surfaces across exposure values and lags (0–56 d) for ATOT, TAVG, and FWI05, respectively. Panels (B), (D), and (F) show fixed-lag slice plots (ATOT, TAVG, and FWI05, respectively), with each row showing RR versus exposure at a single lag: 56 d (top row), 28 d (middle row), and 1 d (bottom row).

The FWI05–lag surface showed a clear cross-over: at short lags (≈0–10 d), drier soils (low FWI05) were associated with elevated relative risk (RR > 1), whereas wetter soils (high FWI05) were linked to reduced risk (Fig.  5E and F). Effects weakened toward intermediate lags (≈20–30 d), where RR was near baseline, and then reversed at longer lags (≈40–56 d), with wetter soils associated with a slightly higher risk. The fixed-lag slices mirrored this pattern. RR declined with increasing FWI05 at 1 day, was near null at 28 days, and increased with FWI05 at 56 days, indicating short-lag sensitivity to dryness and longer-lag sensitivity to wet conditions. Overall effect sizes were modest (≈±1–2% around RR = 1).

The mean temperature surface (Fig. 5C and D) indicated a largely monotonic temperature response across lags. RR was below baseline at cooler conditions and increased steadily with temperature, crossing ∼1.0 near 24–26 °C and remaining ≥ 1 at higher temperatures. The fixed-lag slices were consistently positive in slope at 1, 28, and 56 days, with slightly more substantial increases at longer lags; uncertainty widened at the coolest and warmest extremes, but the central tendency was robust.

The solar radiation–lag surface (Fig. 5A and B) exhibited the opposite trend: relative risk was below baseline at low values of daily accumulated solar radiation (< 15 MJ m^−2^) and increased above 1 for values exceeding ∼23 MJ m^−2^ across the 0–56-day lag window. In fixed-lag slices, relative risk increased with radiation at all three lags, but the increase was more pronounced at 28 and 56 days. At 56 days, for example, relative risk rose from approximately 0.96 at 5 MJ m^−2^ to ∼1.02 at 30 MJ m^−2^.

### Culex pipiens/quinquefasciatus

The univariate TMAX model had the highest weight (*W_i_* = 0.50; AICc = 1,474.82), outperforming VDEF (*W_i_* = 0.31, ΔAICc = 0.95), and SPEI + TMAX (*W_i_* = 0.19, ΔAICc = 1.94). We therefore selected TMAX as the final model; adding SPEI did not sufficiently increase model fit relative to the added complexity.

**Fig. 6.**
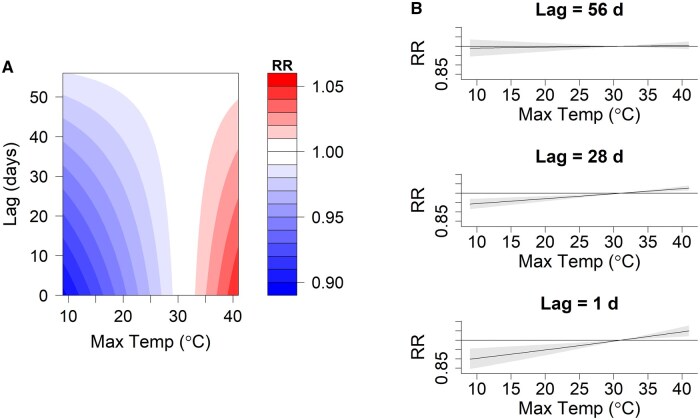
Exposure–lag association for the C*ulex pipiens*/*quinquefasciatus* complex (Diptera: Culicidae) from the selected distributed-lag model including maximum air temperature (TMAX). TMAX = daily maximum air temperature (°C); RR = relative risk (RR = 1 indicates the reference level; RR > 1 higher abundance; RR < 1 lower abundance). A) Contour plot of the RR surface across TMAX values and lags (0–56 d). B) Fixed-lag slice plots showing RR versus TMAX at lags of 56 d (top row), 28 d (middle row), and 1 d (bottom row).

The TMAX lag surface (Fig. 6) exhibited a positive temperature gradient, with a relative risk below baseline at cooler maxima and increasing with higher temperatures. The most substantial effects were observed at short lags, and these were attenuated at longer lags. RR crossed ∼1.0 around 30–32 °C and remained ≥ 1 at warmer temperatures across much of the 0–56-day window. In the fixed-lag slices, RR rose monotonically with TMAX at 1 day, from approximately 0.90 near 10 °C to a value of 1.03–1.04 by 40 °C. At 28 days, the increase was shallower (≈ 0.96 to ∼1.02 across the same range). At 56 days, the curve was close to flat around baseline, with only a slight rise from just under 1.0 at cool temperatures to ∼1.01 at the warmest end.

## Discussion

Mosquito abundance in Norman, Oklahoma, is influenced by a complex interplay of thermal and hydrometeorological factors. Our distributed lag models show that *Ae. albopictus* is best explained by shallow soil moisture at 5 cm depth (fractional water index) and the standardized precipitation–evapotranspiration index (SPEI), indicating that dry soils followed by rewetting combined with wetter conditions at longer lags support higher abundance. For the floodwater mosquito *Ae. trivittatus*, abundance is associated with mean air temperature, shallow soil moisture, and daily solar radiation. Dry soils at short lags and wetter soils at longer lags are consistent with its life history: eggs are laid on ground that periodically dries and then hatch when subsequent rainfall floods these sites, so peak adult abundance often occurs after soils have begun to dry again and temporary pools have receded (Shaman et al. 2002, Mourelatos et al. 2025). Under these conditions, warm, sunny weather may further accelerate larval development and adult activity, leading to higher trap counts several weeks after major rainfall events.

In contrast, the peridomestic *Cx. pipiens*/*quinquefasciatus* appears to respond primarily to maximum air temperature. A notable competing predictor, however, was vapor pressure deficit (VDEF), the difference between saturation and actual vapor pressure, which rises as air warms and relative humidity falls, integrating thermal and moisture conditions (Allen et al. 1998, Ficklin and Novick 2017). Taken together, these patterns suggest that the apparent heat effect may reflect concomitant increases in atmospheric dryness rather than an independent temperature effect (Hagan et al. 2018, Reinhold et al. 2021). Collectively, the species-specific results highlight the importance of soil moisture, drought indices, and solar radiation for the *Aedes* species examined, while the *Cx. pipiens*/*quinquefasciatus* association with temperature likely reflects sensitivity to VDEF.

Our results indicate that mosquito abundance tracks sequences of hydrometeorological conditions that simple temperature–precipitation models do not capture. For *Ae. albopictus* and *Ae. trivittatus*, a characteristic sequence of brief drying followed by rewetting preceded near-term increases, while antecedent wet soils at longer lags set the stage for later surges; accordingly, soil-moisture metrics capture these lagged hydrologic effects. By contrast, *Cx. pipiens/quinquefasciatus* increases tended to follow periods of warm, dry air: maximum temperature performed strongly, and models including VDEF and SPEI were similarly competitive, consistent with a heat association mediated by atmospheric dryness and cumulative moisture balance.

Drought refers to a sustained water deficit relative to normal conditions, not simply a lack of rainfall; indices such as the SPEI combine precipitation and potential evapotranspiration into a standardized *z*-score (Vicente-Serrano et al. 2010, Beguería et al. 2014). Because SPEI integrates water supply and atmospheric demand, it serves as a proxy for the hydrology of mosquito immature habitats, whether containers, catch basins, and ground pools fill and retain water; how often they undergo dry–rewet cycles; and when they flush or concentrate resources. These hydrologic dynamics regulate oviposition, egg hatching, larval survival, and adult emergence (Hoffmann et al. 2020). In our models, SPEI outperformed temperature and rainfall alone, indicating that water stress within the ecosystem is a key driver of mosquito abundance for *Ae. albopictus*. Drought indices provide a physically meaningful way to represent the combined influence of precipitation, evaporative demand, and temperature on the hydrology of mosquito immature habitats, rather than modeling these drivers as separate covariates.

Shallow soil moisture was a key determinant of abundance for the *Aedes* species. In our lagged models, the long- and short-lag terms trace a single sequence, not separate effects: abundance is highest when a brief dry-down follows a wet antecedent spell (elevated shallow soil moisture). Antecedent wetness creates and charges larval habitats, while a short dry period reduces flushing, warms shallow waters and containers, and concentrates organic resources. These conditions favor rapid development and adult emergence (Caldwell et al. 2021). This wet-then-dry sequence accords with the life history of floodwater *Aedes*, whose eggs are deposited on moist substrates, persist through drying, and hatch following subsequent inundation (Shaman et al. 2002, Michigan Mosquito Control Association 2025), and with container-breeding *Aedes*, whose desiccation-tolerant eggs and microclimate sensitivity make emergence responsive to short dry-downs (Day 2016, Westby et al. 2021, Mondal et al. 2025). We note that *Ae. albopictus* is a container-breeding species whose larvae develop in artificial and natural water-holding vessels rather than in soil. However, shallow soil moisture serves as an integrative indicator of the hydrological conditions that govern container water availability: container fill levels, evaporation rates, and dry–rewet cycles are driven by the same precipitation and evapotranspiration dynamics that determine near-surface soil moisture. In this context, FWI05 functions not as a direct mechanism but as a practical, continuously measured proxy for the environmental moisture regime experienced by container habitats. Its pairing with SPEI in the best-supported model further underscores that the relevant signal is the cumulative water balance integrating both water inputs and atmospheric demand rather than rainfall alone. Consistent with this interpretation, a satellite-based study (Chuang et al. 2012) found that microwave-derived soil moisture and surface-water fraction were only weakly correlated with precipitation or humidity yet produced better-fitting models for floodwater mosquitoes than weather-station precipitation, highlighting that hydrological conditions, not rainfall totals alone, drive mosquito abundance.

Solar radiation modulated abundance in complex ways. High insolation warms larval habitats and can accelerate development, a mechanism consistent with the positive associations at multiweek lags observed in our models (Paaijmans et al. 2009). However, intense solar radiation also drives evapotranspiration (Allen et al. 1998), increases desiccation stress in adults and larvae, and can damage larvae through ultraviolet exposure (Villena et al. 2018). Dissolved organic matter and canopy shading can mitigate the effects of UV radiation and desiccation (Evans et al. 2019, Berry et al. 2020). Despite these multiple effects, solar radiation is rarely incorporated into mosquito models. Our finding that solar radiation was a robust predictor of *Ae. trivittatus* abundance, together with other studies showing that sunshine duration and insolation influence *Aedes* mosquito activity (Wang et al. 2020, Cai et al. 2023, Le Goff et al. 2024, Watanabe et al. 2024), indicates that solar radiation merits broader consideration.

Several measures of atmospheric moisture were significant competitors in our models, highlighting that humidity modulates evaporative water loss, egg desiccation, and adult longevity (Kessler and Guerin 2008, Hagan et al. 2018, Holmes et al. 2022). In *Ae. albopictus*, models that paired vapor pressure deficit with temperature or drought indices (e.g., SPEI + VDEF; SPEI + TMIN + VDEF) received appreciable support, suggesting that atmospheric dryness influences abundance. For *Ae. trivittatus*, competing models included dew point temperature (DAVG), mean relative humidity (HAVG), and specific humidity (SH). In *Cx. pipiens/quinquefasciatus*, vapor pressure deficit alone nearly matched the performance of the maximum-temperature model (*W_i_* ≈ 0.31 vs. 0.50; ΔAICc ≈ 0.95). Vapor pressure deficit rises under warm, dry conditions, whereas dew point and specific humidity increase with moisture availability, and these metrics capture different facets of the humidity–temperature interaction (Allen et al. 1998, Lawrence 2005, Ficklin and Novick 2017). Their presence in models across multiple species emphasizes that humidity variables, such as vapor pressure deficit, dew point, relative humidity, or specific humidity, should be considered alongside temperature and precipitation in models of mosquito abundance. This finding is consistent with mechanistic work demonstrating that moisture and temperature jointly influence ectotherm performance and mosquito survival (Yamana and Eltahir 2013, Schmidt et al. 2018, Brown et al. 2023).

The observed patterns across multiple lags suggest that mosquitoes become most abundant following a wet period (providing developmental habitat) that is succeeded by a dry spell (concentrating resources and triggering emergence) (Shaman et al. 2002, Day 2016). Recognizing this environmental sequence can inform control strategies. Monitoring soil moisture and drought indices can signal when a dry spell may precipitate an abundance pulse (Vicente-Serrano et al. 2010, Beguería et al. 2014, Ukawuba and Shaman 2018). Tracking solar radiation and temperature can help anticipate accelerated development (Paaijmans et al. 2009, Steinhoff et al. 2016). Understanding humidity or vapor pressure deficit can help identify periods when adult mosquitoes are more active (Lyons et al. 2014, Holmes and Benoit 2019). Incorporating underutilized variables such as drought indices, soil moisture, solar radiation, and humidity into surveillance models will enhance our ecological understanding and improve situational awareness for mosquito control (Chuang et al. 2012, Lowe et al. 2021, McMahon et al. 2022).

Our study had several limitations. Weather data were sourced from a single Mesonet station approximately 4–7 km from the trap sites, which may not capture all microclimatic variation. However, day-to-day temperature and humidity measurements at our sites closely tracked those of the Mesonet station (see Supplementary Fig. S1), and earlier work comparing microclimate loggers to weather station data in Norman found that model fits were similar for both data sources (McMahon et al. 2022). The microclimate sensors were also limited to temperature and humidity; other variables (e.g., soil moisture, solar radiation) relied on the station. Our study design prioritized temporal depth, 104 weekly collections per site over two field seasons, to support the distributed lag framework, which requires dense, regularly spaced time series. This design reflects a tradeoff with spatial coverage; for comparison, McMahon et al. (2022) sampled 12 sites in Norman at biweekly intervals, providing greater spatial replication but less temporal resolution than the twice-weekly sampling used here. Our design inverted that tradeoff, favoring the temporal density needed for distributed lag estimation over spatial coverage. The four sites were selected to span a range of land cover types within Norman (mixed woodland, urban park, residential yard, and riparian/impervious surface), and the site-level random intercepts capture differences in baseline abundance across these environments. However, the small number of sites limits the ability to generalize findings across broader spatial scales. With a larger number of sites, longer time series, and complementary trap types (e.g., gravid traps for Culex) (Reiter 1983, Kline 2006), future studies could better characterize spatial variability and species composition. We also constrained our distributed-lag models to linear exposure–response functions, a pragmatic choice for a smaller dataset; larger datasets could support testing nonlinearities and interactions between temperature and humidity using distributed lag nonlinear model frameworks (Gasparrini et al. 2010, Gasparrini 2011). Additionally, more complex temporal correlation structures [e.g., AR(1) residuals] could be explored in future analyses with longer time series (Wood 2017, Clark and Wells 2023).

By moving beyond a temperature–precipitation paradigm, this study demonstrates the value of integrating thermal, hydrological, and atmospheric drivers to interpret mosquito dynamics under current and future climates. In addition to temperature and rainfall, soil moisture, drought indices (e.g., SPEI), solar radiation, and humidity capture ecological processes that are essential to mosquito population dynamics, such as floodwater hatching after rainfall, the effects of soil wetness on larval survival, and desiccation stress on adults. Our modeling approach focused on model comparison and is not intended to be predictive; however, the observed patterns provide operational guidance. Monitoring soil moisture and drought to anticipate abundance pulses, as well as tracking solar radiation and humidity to gauge potential shifts in activity, can help refine local risk assessments. Future development of mechanistic models should couple thermal biology with hydroregulation, photoperiodic responses, and microclimate measurements and incorporate human behaviors (e.g., water-storage practices) and pathogen surveillance. Such ecology-informed approaches will be increasingly crucial for forecasting mosquito-borne disease risks as climate variability intensifies.

## Supplementary Material

tjag102_Supplementary_Data
