# Supplementary material for: Hydrometeorological drivers of mosquito (Diptera: Culicidae) abundance in an urbanized region of central Oklahoma
Source: J Med Entomol. 2026 Jul 10;63(4):tjag102. doi: 10.1093/jme/tjag102 (PMC13354607; doi:10.1093/jme/tjag102)
Supplement: tjag102_Supplementary_Data [file tjag102_supplementary_data.docx]

**Supplemental Materials**


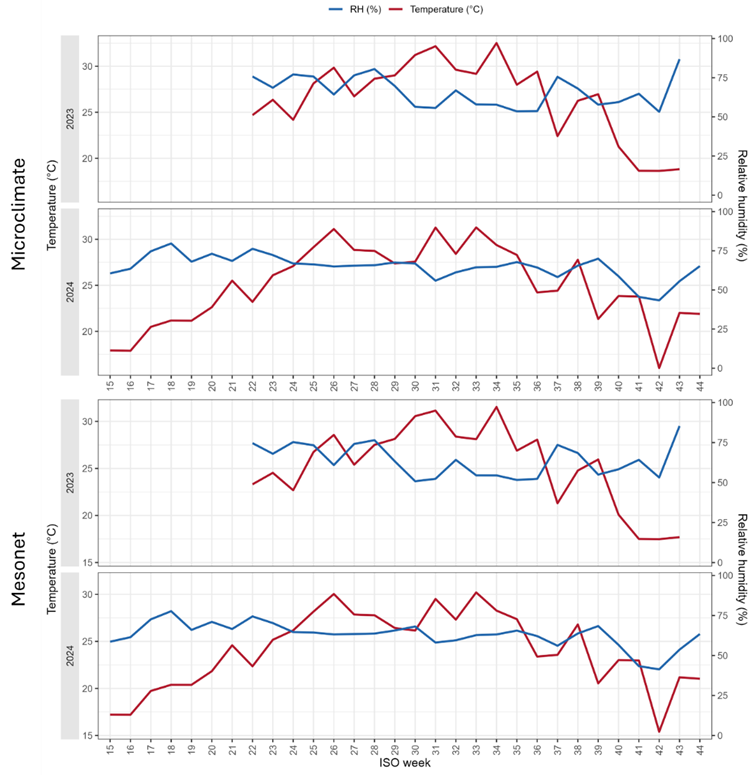


**Figure S1:** Weekly means by ISO week for Microclimate (top two panels) and Weather Station (bottom two panels) in 2023 and 2024. Red lines show temperature (°C; left axis) and blue lines show relative humidity (%, right axis). Microclimate values are site-averaged daily means aggregated to ISO weeks; Weather Station values are station daily means aggregated to ISO weeks. Only weeks present in both datasets are plotted, so the timelines align across years.

**Table S1.** Cumulative and lag-specific relative risk (RR) estimates from distributed lag models for each predictor in the selected models.

| **Species** | **Predictor** | **Contrast** | **Cumulative** | **Lag 1 d** | **Lag 28 d** | **Lag 56 d** |
| --- | --- | --- | --- | --- | --- | --- |
| ***Ae. albopictus*** | FWI (5 cm) | 0.90 vs 0.50 (ref) | 0.909 (0.717–1.153) | 0.990 (0.981–0.999) | 0.998 (0.994–1.002) | 1.007 (0.999–1.015) |
|  | SPEI | 0.75 vs 0.00 (ref) | 0.979 (0.808–1.186) | 0.991 (0.987–0.995) | 1.000 (0.996–1.003) | 1.008 (1.002–1.015) |
| ***Ae. trivittatus*** | FWI (5 cm) | 0.90 vs 0.50 (ref) | 0.646 (0.497–0.839) | 0.981 (0.970–0.993) | 0.992 (0.988–0.997) | 1.004 (0.992–1.016) |
|  | Mean temperature | 29.00 vs 24.96 (ref) | 1.356 (1.135–1.620) | 1.003 (0.994–1.012) | 1.005 (1.002–1.008) | 1.008 (0.998–1.017) |
|  | Solar radiation | 25.00 vs 21.79 (ref) | 1.927 (1.361–2.728) | 1.009 (1.000–1.018) | 1.012 (1.005–1.018) | 1.014 (1.005–1.024) |
| ***Cx. pipiens/quinq.*** | Max temperature | 36.00 vs 31.06 (ref) | 2.145 (1.480–3.107) | 1.024 (1.010–1.038) | 1.014 (1.007–1.020) | 1.002 (0.992–1.013) |

**Note:** RR is evaluated at the 75th percentile of each predictor relative to the centering value (median for temperature variables, 0.5 for FWI, 0 for SPEI). The reference value (ref) is the centering point at which RR = 1.0. Cumulative RR represents the overall effect integrated across all 56 lag days. Lag-specific values show the effect at individual lags (1, 28, and 56 days). Values in parentheses are 95% confidence intervals. For predictors with opposing short- and long-lag effects (e.g., FWI, SPEI), the cumulative RR may understate the magnitude of lag-specific associations. RR estimates are from univariate models (one predictor per model), consistent with the contour and slice plots presented in Figures 4–6.
